# The impact of violence on healthcare workers' mental health in conflict based settings amidst COVID‐19 pandemic, and potential interventions: A narrative review

**DOI:** 10.1002/hsr2.920

**Published:** 2022-11-06

**Authors:** Aiman Rija, Zarmina Islam, Wajeeha Bilal, Khulud Qamar, Shazil Ahmed Gangat, Samina Abbas, Hania Tul Mirha, Parvathy Mohanan, Zainab Syyeda Rahmat, Sean Kaisser Shaeen, Selma Nihel Klouche Djedid, Mohammad Yasir Essar, Rahul Kashyap

**Affiliations:** ^1^ Faculty of Medicine, Dow Medical College Dow University of Health Sciences Karachi Pakistan; ^2^ Department of Medicine CMH Lahore Medical College Lahore Pakistan; ^3^ Medical University Sofia Sofia Bulgaria; ^4^ Faculty of Medicine University of Tlemcen Tlemcen Algeria; ^5^ Kabul University of Medical Sciences Kabul Afghanistan; ^6^ Critical Care Medicine Mayo Clinic Rochester Minnesota USA

**Keywords:** abuse, conflict, COVID‐19, healthcare workers, humanitarian crisis, mental health, violence

## Abstract

Healthcare workers (HCWs) have faced an increased amount of mental health struggles amidst the COVID‐19 pandemic. However, those in conflict‐based settings with fragile healthcare systems meet additional challenges. This study reviews violence, conflict and mental health among HCWs in five countries: Syria, Palestine, Yemen, Afghanistan and Lebanon. Our study reports that HCWs are targeted by violence, bombings, mistreatment and different forms of abuse, including verbal and physical. With the additional burdens of the pandemic including prolonged working hours, limited resources and insufficient humanitarian aid, the healthcare workers fall victim to increased levels of burnout and mental illnesses. The situation leads to dire consequences on their personal lives and professional development, compelling them to quit their job or country all together. Although healthcare workers remain resilient in these conflict‐based settings, immediate interventions are required to prevent violence against them and cater to their rapidly declining mental health.

## 1 INTRODUCTION

Global prevalence of elevated mental health struggles is evident since the onset of the pandemic.^1^ A cross‐sectional study comprising 465 young individuals with no history of mental health illnesses, depicted more than half of them experiencing feelings of severe anxiety and decline in the quality of life during the early phase.^2^ A definite and mild impact of the pandemic on physical and psychological stress was thus reported.^2^ Several studies and reviews have confirmed healthcare workers (HCWs) experiencing greater instances of stress, burnout and mental illnesses.^3^, ^4^, ^5^ This is associated with increased workload, uncertainty, fear, frustration, lack of rest and personal protection, and prolonged working hours during the pandemic.^6^ Acute stress faced by HCWs has transitioned into chronic stress during the two years of the prolonged pandemic, as depicted by a longitudinal study centered in two hospitals of Italy.^4^ Since the study evaluated the mental health of HCWs during each of the four successive waves of the pandemic, the frequency of sleep problems and anxiety reported during the first phase gradually decreased over time. However, the high levels of depression, workload and perceived stress persisted. Dealing with the Anti‐Vaccine Community accounted for an added occupational stressor for the HCWs during the fourth wave, as HCWs found themselves at the receiving end of their threatening and aggression.^4^ A systematic review including more than 16,000 HCWs from 15 studies reported exacerbation of violence towards HCWs during COVID‐19, and mental health problems to be substantially higher in HCWs who faced Workplace Violence than those who did not.^5^ The HCWs face greater challenges in conflict‐based settings, such as Syria,^7^ Palestine,^8^ Yemen,^9^ Afghanistan,^10^ and Lebanon.^11^ Political instability, economic burden, limited resources, lack of safety and a fragile healthcare system, predispose a complex ordeal difficult for HCWs to navigate through.^10^ In such settings, the healthcare system has also been repeatedly targeted due to the ongoing political turmoil, costing lives of many HCWs and destruction of several healthcare facilities.^12^, ^13^, ^14^ The conflict situation along with the added burden of violence and mistreatment from the masses,^15^ worsens the well being of HCWs, making them increasingly susceptible to mental illnesses. As a result, HCWs have also been reported to consider leaving their job or country in an attempt to escape these helpless situations.^16^

### 1.1 Aims of review

This review aims to assess current literature on the impact of violence on the mental health of HCWs for the following conflict‐based settings: Afghanistan, Lebanon, Palestine, Syria, and Yemen. These countries were selected based on their timely relevance within the context of the COVID‐19 pandemic. Moreover, the review focuses on providing practical recommendations to protect the healthcare system from conflict, to address the mental health needs of such settings and alleviating the struggles of HCWs.

## 2 Methodology

A search was conducted on PubMed database and Google scholar from January, 2020 to April, 2022, related to the following key terms: “Yemen,” “Afghanistan,” “Syria,” “Lebanon,” “Palestine,” “Mental Health,” “Healthcare Workers,” “Qualitative,” “Workplace Violence,” “conflict,” “violence,” and “COVID‐19.” Additionally, the web was searched for relevant information surrounding each conflict setting including news broadcast channels such as Al‐Jazeera, and international entities such as World Health Organization, Human Rights Watch, United Nations, and Doctors Without Borders. Articles were selected on the basis of the inclusion criteria, which included cross‐sectionals, qualitative studies, systematic reviews, case studies, and reports from various organizations published in English. Exclusion criteria includes articles published in languages other than English. Qualitative and Cross‐sectional Studies were included in the table, and researchers cross‐checked the data to avoid any discrepancies.

## 3 RESULTS

### 3.1 COVID‐19 and violence on HCWs

The onset of COVID‐19 has exponentially demanded more work hours, staff, and patient care quality from HCWs across the globe regardless of how developed and efficient the hospital had been before the pandemic. In addition to these hardships, the families of patients and general communities have shown ill‐mannered behavior instead of appreciation towards HCWs. ^17^ This leads to violence against HCWs which stems from multifactorial conditions that include anxiety, fear of worsening health status of patient, financial instability, expectations of speedy care from HCWs, lack of awareness, stigmatization.^18^ The acts of violence against HCWs have surged in the pandemic, these include chaos due to political turmoil, distrust from citizens, attacks from visitors and more factors that revolve around negative public perception of health care systems, in general. Ramzi ZS et al in their recent systematic review and meta‐analysis, concluded violence against HCWs to have risen during the pandemic, with the prevalence estimated to be 47% ranging from verbal to physical violence.^19^ The study included 17,201 medical staff and the prevalence of workplace violence statistics was irrespective of sample sizes and the age of HCWs.^19^ The International Committee of Red Cross (ICRC) reported total 611 cases where HCWs faced violence, harassment and stigmatization including 20% being physical assault, 15% being discriminatory violence and 15% being verbal abuse.^17^ These harsh challenges have drastically affected the mental health on HCWs all over the world to the limit of exhaustion, burnout, stress and overwhelmedness. Bitencourt et al. states that HCWs exposed to violence during COVID‐19 have scored higher (statistically significant) in the Generalized Anxiety Disorder‐Grade 7 (GAD‐7) scale compared to those who have not experienced violence in workplace.^20^

### 3.2 Violence and mental health of HCWs in conflict‐based settings

Countries under conflict settings have faced with worse challenges than those without political disruption. For HCWs, working for public health has been extremely dangerous in conflict settings as stated by SHCC.^17^ According to Humanitarian Outcome, HCWs face violence through damage of health facilities and harm from the patient, their families and general community.^17^ Bou‐Karroum et al. states in their scoping review that 63% of 136 articles mention violence against HCWs as an alerting concern among conflict‐ based settings.^21^ The acts of violence have become life‐threatening for HCWs, hence deteriorating their mental health. Fear of life, anxiety, PTSD, and depression has been widespread, seeking mental health services as well is not feasible due to lack of resources, stigmatization, and inefficient therapeutic measures. These reasons may prevent HCWs from prioritizing their own well being leading to inability to perform clinical care to the best of their abilities.

### 3.3 Syria

Although the warfare in Syria has forced the entire population to experience painful processes of displacement, symptoms of mental disorders and feelings of overwhelming distress,^22^ the impact of violence on healthcare workers has been gruesome. Attacks on the healthcare system have resulted in the death of more than 900 HCWs and damage to 600 healthcare facilities since 2011.^7^ In 2018 alone, 194 attacks on healthcare facilities and ambulances have been verified.^23^

Amidst the political turmoil and the onset of COVID‐19 pandemic, HCWs in Syria face the brunt of repeated violence, lack of resources and a fragile healthcare system.^7^ Workplace violence towards HCWs was studied in the public hospitals of Syria in 2021,^16^ and the cross‐sectional study reported almost 85% of participants reported exposure to workplace violence in the past year. Furthermore, 930 deaths of medical professionals have been reported in Syria from 2011 through March, 2021.^24^ According to a study published in 2021, more than 80% of the doctors reported to have undergone physical and verbal workplace violence in the span of one year.^16^ There is a need for resources to study long term outcomes of violence on mental health among HCWs. Syria would be the one the first places to conduct such study.

### 3.4 Palestine

Palestinians have faced decades of conflict, traumas and social distress, leading to anxiety and PTSD being the most common mental disorders among the population.^25^ With the onset of the pandemic, the healthcare system of Palestine faced a major setback, and the mental health of the healthcare workers deteriorated even further.^8^ Healthcare Professionals in Palestine have been reported to feel isolated, anxious and under‐appreciated during the COVID‐19 response.^26^

Moreover, healthcare workers in Palestine have also been exposed to work place violence, including verbal and non‐verbal violence.^27^ A cross sectional study centered around emergency departments in Palestinian hospitals reveals that nonphysical violence mostly included verbal abuses (69.8%), threats (48.4%), and sexual harassments (8.6%).^15^ Furthermore, Gaza's fragmented healthcare system found itself in shambles with repeated instances of war.^13^ While hospitals were overwhelmed with injured people, two of Gaza's most prominent doctors in Gaza's coronavirus task force, were also killed.^14^

### 3.5 Yemen

The ensuing war in Yemen has fragmented the country and injured, killed and displaced thousands of civilians.^28^ It is estimated that an average 25‐year‐old in Yemen has already lived through 14 armed conflicts.^28^ Healthcare facilities in Yemen have been targeted at least 120 times as a result of conflict.^9^ In 2020, An‐Nasr Hospital in Yemen was attacked twice in 1 week.^29^ The onset of the COVID‐19 pandemic has frightened HCWs who continuously provide clinical care despite poor preparedness and weak healthcare infrastructure.^30^ During year 2020, The Safeguarding Health in Conflict Coalition (SHCC) identified 81 incidents of damage to the healthcare facilities in Yemen as a result of violence and 35 incidents in year 2019 and numerous HCWs were killed and injured as a result.^29^ Additionally, HCWs found themselves at the receiving end of violence from communities due to misinformation about COVID, transfer to quarantine centers, frustration on restrictions and anger at the loss of loved ones.^31^ Such ordeals negatively impacted the mental health of HCWs. A survey conducted in year 2020 reported about 70% Yemeni HCWs feeling moderate‐severe stress.^32^

### 3.6 Afghanistan

Due to the persistent conflict in Afghanistan, it is reported that more than 85% of the population has personally experienced or witnessed at least one traumatic event in their life.^33^ Afghanistan's under‐resourced healthcare system is repeatedly subjected to violent attacks as a result of political instability.^34^ Due to violence and conflict, 24 health facilities were damaged in the year 2017 and 154 HCWs were injured.^35^ This further led to 150 health facilities forcibly closed temporarily due to insecurity and violence, compromising access to the healthcare to 3 million people.^35^ The studies have mentioned total 75 attacks on HCWs in 2019 and an attack on a maternity hospital in 2020.^34^ During COVID‐19, the impact of violence on HCWs and the working conditions were significantly exacerbated by limited staff, restricted humanitarian aid, prolonged working hours, and suboptimal preparedness.^10^ HCWs face greater instances of exhaustion, stress and burnout, which negatively influence their mental health.

### 3.7 Lebanon

Data from Lebanon depicts severe levels of distress among its population.^36^ Lebanon is under‐resourced to meet the mental health needs of its population as only 5% of the total governmental health budget is allocated to it.^11^ HCWs struggle to manage the deteriorating conditions and meet the exceeded burden during COVID‐19, thus they face greater stress and lesser motivation to continue their jobs.^11^ The experiences of HCWs have worsened due to the healthcare system being targeted with violence and threats.^37^ At one instance, four out of 15 HCWs interviewed reported being subjected to verbal or physical abuse by the population.^37^ A survey conducted after the onset of the pandemic revealed 61% of the HCWs experiencing moderate to severe symptoms of anxiety, and approximately 50% experiencing sleep disturbances.^38^ With the emergence of COVID‐19 outbreak, the country is thought to be under a “two‐in‐one crisis.”^38^ It is evident that HCWs face high risks of burnout and emotional exhaustion in this population,^38^ and being violently targeted^39^ further pushes their mental health off the edge.

## 4 DISCUSSION

Based on the literature review, violence was found to have a strong association with the rapidly deteriorating mental health of the healthcare workers in conflict‐based setting across the world. We have detailed our discussion relevant to each country and Table 1 highlights the studies focusing violence on HCWs in conflict‐based settings and its effect on their mental health.

**Table 1:** This table highlights studies relevant to violence on HCWs in Syria, Palestine, Yemen, Afghanistan, and Lebanon

Author	Country	Year	Study/objective	Population	Study type	Main findings	Limitations
Abdelrahman et al.^13^	Syria	2021	To explore the effect of conflict on healthcare workers in Syria	*N* = 82 healthcare workers	Qualitative	High degree of risk (71%) Traumatic experience (87%) Stress (85%)	The study does not represent areas of Syria controlled by the government
							It is majorly a representation of male healthcare workers. The sample size was small and sampling may be prone to bias due to lack of random selection of participants
Mohamad et al.^16^	Syria	2021	To explore workplace violence toward resident doctors in public hospitals of Syria	*N* = 1127	Cross‐sectional	Verbal violence faced (84.74%) Exposed to physical violence (19.08%) Workers experienced violence related injuries (70%) Association of workplace violence with the mental health of workers (violence is positively associated with psychological stress and depressive symptoms)	The data is limited by specific geographical regions as well as specific hospitals within Syria
Bahaa Aldin Alhaffar et al.^40^	Syria	2021	To assess the psychological Effects on Healthcare Workers in Syria During COVID‐19	*N* = 584	Cross‐sectional	workers experienced poor sleepy quality (80%) severe stress disorder (27.9%) generalized stress disorder (70%)	The data collected was based on an online survey, thus lack of internet connection placed limitations on the result as the questionnaire would be inaccessible to some
Saeedi et al.^27^	Palestine	2021	To study the potential impact of verbal and nonverbal miscommunications between the patients and healthcare workers upon workplace violence from the patients' perspectives	*N* = 505 patients and previously hospitalized patients	Cross‐sectional	7.2% of the study population reported participating in nonverbal violence and 19.6% participated in verbal violence against healthcare workers	The patients who refused to participate in the study could be the ones who might be a greater contributor to the violence against healthcare workers
Hamdan and Hamra^42^	Palestine	2017	To assess burnout levels among health workers in Emergency Departments (Eds) in Palestinian hospitals, and their associated risk factors including workplace violence	*N* = 444 workers participated (161 nurses, 142 physicians and 141 administrative personnel)	Cross‐sectional	Results showed high levels of burnout among EDs workers; 64.0% suffered from high emotional exhaustion, 38.1% from high depersonalization and 34.6% from low personal accomplishment Exposure to physical violence was significantly associated with high degrees of burnout but no association was observed with regard to exposure to verbal violence Burnout was significantly associated with workers' intention to leave work at EDs	This study adopted a cross‐sectional design, thus it's difficult to draw proper conclusions on causal effect relationships. The study data is self‐reported and might have produced a social desirability bias in the responses of participants

Hamdan and Abu Hamra^15^	Palestine	2015	To study the Workplace Violence experienced by ED workers in Palestinian hospitals, including the types of violence, their perceived causes and consequence	*N* = 444 participants (161 nurses, 142 physicians, and 141 administrative personnel)	Cross‐sectional	76.1% experienced a type of Workplace Violence in the past 12 months: 35.6% exposed to physical and 71.2% to nonphysical assaults (69.8% verbal abuses, 48.4% threats, and 8.6% sexual harassments) Perpetrators were mainly patients' families/visitors	The possible reluctance of participants to report exposure to violence because of fear of stigmatization, underreporting and recall bias

Elnakib et al.^44^	Yemen	2020	To understand how the conflict in Yemen impacted daily lives of HCWs, and their coping mechanisms	*N* = 43 interviews of HCWs	Qualitative	Daily stressors prevented HCWs from carrying out their duties at work High levels of insecurity, and violence at facilities and residences have increased depression and anxiety HCWs express feelings of inadequacy and helplessness Financial barriers such as suspension of pay, and shortage of essential medical supplies have influenced quality of care A meaning‐focused approach is used to cope in a healthy way, that increases self‐efficacy Religion, a duty to serve, and innovation play a positive role in coping with the workload	Limited by experiences of HCWs late 2018, the data does not reflect on HCWs who left service, and since the initial study design was not meant for this study, data collection issues remain
				2 reproductive healthcare providers, 16 maternal and newborn healthcare, 3 immunization specialists, 5 child healthcare, 5 nutrition specialists and 12 healthcare managers or generalists			

Najafizada et al.^50^	Afghanistan	2014	To describe the CHW program, explore the gender dynamics of the workforce, and identify facilitators and challenges to the program	*N* = 55 key informants (25 CHWs, 9 CHW supervisors, 4 CHW trainers, 6 managers, and 11 policy makers)	Qualitative	More female CHWs than male Gender hierarchy: At higher levels, the ratio of women to men diminishes Female CHWs accomplished task with greater ease than male counterparts CHWs are driven by internal motives to volunteer, but may leave for better opportunities CHWs express receiving blame, ill‐treatment and low financial support and drug allocations	Small sample of 16 health posts, and the inability to survey health posts of insecure provinces
Deeb^51^	Lebanon	2003	Workplace violence in the healthcare sector; any type of harassment, abuse, aggressive behavior or attitude, and mistreatment whether it is physical or psychological/emotional	Physical, *n* = 1009 Verbal, *n* = 1008	Country Case Study including qualitative and quantitative components	The dimension of the workplace violence showed that the prevalence of verbal abuse was the highest (41%) followed by bullied/mobbed (22.4%), physically attacked (6%), racially harassed (4.9%) and sexually harassed (2.4%) For physical violence, the patient or a relative was the main perpetrator, while for bullying and verbal abuse, sexual and racial harassment, it was mainly either a staff member or a colleague or a manager. It happens most of the time for all types of violence inside the institution	
				Bullying, *n* = 997			
				Sexual, *n* = 983			
				Racial, *n* = 981			

Abbreviation: HCWs, Healthcare workers.

### 4.1 Syria

Syria has been in a state of violence for over a decade now, with healthcare workers in specific being targets of both judicial and nonjudicial means of persecution. As presented in a particular study, 14.71% have experienced violence, 42.37% have faced civilian bombardment and blockade, 35.59% were detained or arrested and 15.25% experienced attacks on health facilities.^13^ Workplace violence against healthcare workers is very prominent in Syria due to the ongoing conflict as well as the burden faced on the healthcare system because of the pandemic.

Another study states that 84.74% of resident doctors face verbal violence, whereas 19.08% face physical violence.^16^ The findings also noted that male resident doctors face a significantly higher frequency of violence in comparison to females.^16^ Violence has greatly impacted the mental well‐being of the workers, along with affecting their job performance and job satisfaction.^16^ Many healthcare workers have experienced a rise in anxiety and depression along with poor sleep.^16^

Based on results delivered by a study, 15.9% of the practitioners worked 10–12 h daily.^40^ Along with violence and staff shortage, the healthcare system also lacked proper equipment and resources. The study shows 43.3% of healthcare workers claim their facility does not provide the required equipment to protect them against acquiring COVID.^40^ As a result of the current condition, 72.4% of workers had poor sleep quality and 17% showed severe stress disorder.^40^ The ongoing Syrian conflict has resulted in the displacement of millions of Syrians, while making the lives of those still in the country challenging.^41^ The dire conditions surrounding the healthcare system contribute to the rapid decline of the mental health of HCWs, compelling up to 70% HCWs to leave the country.^7^

### 4.2 Palestine

Palestinians are not only battling with a pandemic but also with Israeli occupation and violence. The fear of mass bombings and killings further exacerbates feelings of anxiety and depression caused by the pandemic. HCWs are also subjected to this violence, two well‐known doctors were killed due to bombings,^14^ and the only COVID‐19 testing laboratory in Gaza was destroyed due to an airstrike.^14^

The healthcare workers in Palestine have been reported to feel exhausted fighting alone in the pandemic, in the absence of the support that they need.^26^ Previous studies have provided evidence for high levels of burnout among HCWs.^42^ Exposure to violence, especially physical violence at workplace is one of the factors associated with such levels of burnout.^42^ A study has reflected violence on about 75% healthcare staff included in the study, mainly inflicted by the patients' families and visitors.^15^ The violence adopted many forms; physical and nonphysical, including verbal abuse, threats, and sexual harassment.^15^

In another study conducted in 2021, 7.2% of the study population reported participating in nonverbal violence and 19.6% participating in verbal violence against the healthcare workers.^27^ Such events are thought to be under‐reported and are majorly attributed to the absence of appropriate consequences against them.^15^ This lack of a healthy and safe working environment negatively affects the mental health of HCWs, their patient care and willingness to continue their job.^15^

### 4.3 Yemen

Since the conflict first began, healthcare facilities have been targeted at least 120 times according to Human Rights Watch, and 100 attacks in 2017 alone (reported from World Health Organization.)^9^ Between 2015 and 2016, 93 attacks on hospitals were reported, and 102 facilities were partially or completely damaged amounting to 4.65 attacks per month.^43^ Limited functionality of healthcare facilities coupled with abuse from both patients and political militias have made it difficult for HCWs to carry out their work.^44^ This has lowered workplace efficacy, and disempowered HCWs as reported in Elnakib's study.^44^

Lawlessness and insecurity have made violence accessible to militias, and hence accounts of raids, shelling, and gunfire at residences of HCWs or their family members have been reported. In the face of delivering healthcare services, HCWs lose their own lives as they become targets during travel, or upon arrival. Witnessing or hearing such traumatic incidents impacted mental wellbeing. There have even been instances of HCWs feeling like they must relive those traumas or violent encounters. Some HCWs even expressed shame at their incapacity and helplessness, which has directly contributed to self‐doubt.^44^ Unsafe working conditions, fears of threats or harassment, and roadblocks when leaving for work in the morning are some of the challenges HCWs face. This has resulted in greater distress for HCWs who are emotionally and physically exhausted before they even reach their workplace.^44^

Paralleling the spread of COVID‐19, armed conflict intensified in Aden following April 2020, which is expected to have worsened conditions for HCWs in this area.^45^ Geographical differences must be considered for the case of Yemen as varying levels of political conflict influence availability of aid, and level of violence experienced.

### 4.4 Afghanistan

According to a report published in 2019, 140 health facilities in Afghanistan were reported to be forcefully shut down by armed groups.^46^ In May 2020, a militant attack was directed at a government‐run Maternity Hospital in the Afghan Capital, claiming 24 lives.^47^ 15 mothers, three of whom were in the delivery room, two young boys and a local midwife were among those killed.^48^ Three HCWs and two new‐born babies were injured.^48^ Since, Médecins Sans Frontières (Doctors Without Borders) were assisting the hospital in healthcare provision, an attack planned at them predisposed them to increased vulnerability and harm, forcing them to exit the hospital practice.^49^ Community Health Workers have also expressed ill‐treatment at the hands of both, the healthcare system and the general population. They are viewed to not have best interests at heart and are blamed for the suboptimal health services and an ineffective healthcare system in rural settings.^50^

During the pandemic in 2020, Action on Armed Violence monitoring project reported Afghanistan to be the worst impacted country globally,^12^ due to numerous civilian casualties resulting from explosive weapons. With 50% prevalence of psychological distress, mental health ordeals impede normal functioning in about 40% of the population.^33^ While such extremely violent state of affairs and a pandemic, put HCWs at the risk of losing their health or lives, it also deteriorates their mental health to a great degree. Practitioners have no choice but to function under constant fear of personal and family safety. They can find it extremely challenging to cope with work, travel and provide healthcare to the masses, while their minds continue to stay on guard.

### 4.5 Lebanon

In particular, HCWs have been victimized by violence and harassment. Especially when hospitals could not admit any new patients amidst the pandemic, some healthcare workers became targets of violent attacks.^37^ While doing their jobs, patients or their families threatened and physically assaulted the healthcare providers.^37^ In an interview with Human Rights Watch, Abou Sharaf, Head of the Lebanese Order of Physicians, stated that at least one serious attack on a physician occurs every month.^37^ An emergency room doctor at a private hospital in Saida reported several occasions of patients' families attacking healthcare workers or damaging the intensive care unit after not being allowed to visit their sick relatives.^37^ Apparently, one incident involved people smashing the glass barrier and attacking caregiver.^37^

Gunmen have used hospitals to fire weapons, drawing fire in return.^39^ Physicians have been forced to administer medical treatment at gunpoint and several doctors have been killed at roadblocks.^39^ A case study conducted in Lebanon on Workplace Violence in the Health Sector states verbal abuse to be the most prevalent (41%) among the dimensions of workplace violence followed by bullying/mobbing estimated to be less than 25%.^51^ Statistics of physically attacked, racially and sexually harassed were less than 10%.^51^ A patient or a relative likely perpetrated physical violence, whilst for bullying, verbal abuse, and racial and sexual harassment, it was usually a coworker or a manager.^51^

### 4.6 Efforts and recommendations

#### 4.6.1 Blast prevention

There are various ways governments and organizations have worked to stop or reduce the rising number of incidents of violence against healthcare workers. (Table 2 provides a summary of recommendations to reduce violence against HCWs.) The ICRC has suggested and implanted preventative strategies that seem to have done well in their respective fields. One method which aims to protect healthcare workers from physical attacks in their working environment specifically, is by having film installed on hospital windows to prevent discharged explosive devices from turning the glass of the same windows into potentially deadly high‐speed projectiles.^52^ Additionally, with respect to transit to and from the workplace, individuals have discussed and negotiated with locals and local militants to allow for the safe passage of ambulances through various checkpoints and areas.^52^ Furthermore, adding additional security measures as well as an increased number of staff have been used as a means to reduce violence against healthcare workers. Reducing the amount of time patients remain in the hospital setting decreases the interaction time between patients and healthcare workers hence allowing for less chances of said patients becoming violent against said healthcare workers.^53^

**Table 2:** Summary for recommendations to prevent/decrease violence on healthcare workers' in conflict based settings for better mental health

Recommendation	Impact
1. Installing films on hospital windows to prevent explosive devices damage	Decrease physical trauma and violence against HCWs and patients
2. Security staff	Guarantee safety of HCWs
3. Peer support	Increase mental health support for HCWs
4. Hospital administrative polices	Avoid violence against HCWs
5. Clinical research and registries	Provide more evidence on violence against HCWs

Abbreviation: HCWs, Healthcare workers.

#### 4.6.2 Mental health support

Several countries have adopted interventions and introduced programs to cater to the growing mental health needs of their population. Mental health hotlines are operational in Afghanistan^10^ and Lebanon.^11^ Awareness sessions and training programs have played a major role in breaking the stigma and encouraging people to seek support. In Afghanistan, WHO and the Interagency Standing Committee produced mental health support guidelines, which were also translated in local languages to expand access.^10^ In Palestine, attempts at fostering mental health included distribution of educational material^8^ and training provided by the Gaza Community Mental Health Program.^8^ In Syria, the Battle Buddies program comprising of peer‐to‐peer support groups, and the Problem Management Plus program, aimed to provide mental interventions and support to the HCWs.^40^

#### 4.6.3 Hospital administrative polices

To alleviate the struggles of HCWs because of violence, their safety should be ensured regardless of their location, political affiliation, or the civil populations they serve.^13^ At the healthcare system level, HCWs must be shielded from aggression and violence through introduction of additional policies and implementation of the existing legislation.^16^ In a study conducted in Syria,^16^ the healthcare worker respondents recommended restricting visitors' access, devising a rigorous violence reporting system, and developing better security coverage through security guards and cameras, to ensure personal and security satisfaction in a workplace environment. Workers employed in a healthcare setting should be enrolled in violence management educational programs, in an attempt to raise awareness and shed light on dealing with aggression.^16^ Structuring a detailed violence prevention program for the HCWs would assist them in gaining vital skills necessary for conflict management, effective communication and de‐escalation of an argument or aggressive encounter.^16^

#### 4.6.4 Peer support

Additionally, the introduction of support programs and groups amongst the healthcare fraternity would help foster unity and mental health of HCWs in times of crisis. Provision of adequate resources, delivering timely salaries and introduction of tele‐medicine programs are some of the interventions that would help ease the HCWs' workload and in turn, would contribute to an improvement in burnout statistics and their overall mean health. Governments must ensure safety measures for HCWs during work and transportation, so that they are not subject to conflicts and retaliation.^13^ Lastly, the international community must prioritize voicing concerns for the HCWs working in conflict‐ based settings and holding concerned authorities accountable^13^ for maintaining safety standards in healthcare settings. This combination of demonstrating global empathy and provision of continued humanitarian aid,^10^ like vaccine supply and monetary support, could prove to be instrumental in transforming work experiences and mental health of HCWs struggling in conflict‐based settings.

#### 4.6.5 Clinical research and registries

The data coming out from each conflict setting is fractured and non‐harmonized. The effect on HCWs mental well being is not well studied and its impact on patient clinical care is even less studied. The need of the hour to develop and sustain near real time clinical registries^54^, ^55^, ^56^, ^57^ to capture harmonized clinical data from hospitals, HCWs and patients.

## 5 CONCLUSION

In conclusion, HCWs in conflict‐based settings fall target to multi‐layer of violence and mistreatment, subsequently increasing their susceptibility to mental illnesses. In spite of countries' specific challenges, HCWs also have demonstrated remarkable levels of resilience. This review article sheds light on the possible interventions and the urgent need for their timely implementations to shield the healthcare system from violence and improve the mental well‐being of HCWs in conflict‐based settings.

## AUTHOR CONTRIBUTIONS

**Aiman Rija**: Writing – original draft. **Zarmina Islam**: Writing – original draft. **Wajeeha Bilal**: Writing – original draft. **Khulud Qamar**: Writing – original draft. **Shazil Ahmed Gangat**: Writing – original draft. **Samina Abbas**: Writing – original draft. **Hania Tul Mirha**: Writing – original draft. **Parvathy Mohanan**: Writing – original draft. **Zainab Syyeda Rahmat**: Writing – original draft. **Sean Kaisser Shaeen**: Writing – original draft. **Selma Nihel Klouche Djedid**: Writing – original draft. **Mohammad Yasir Essar**: Conceptualization; supervision; writing – review and editing. **Rahul Kashyap**: Writing – review and editing.

## CONFLICT OF INTEREST

The authors declare no conflict of interest.

## TRANSPARENCY STATEMENT

The lead author Mohammad Yasir Essar affirms that this manuscript is an honest, accurate, and transparent account of the study being reported; that no important aspects of the study have been omitted; and that any discrepancies from the study as planned (and, if relevant, registered) have been explained.

## Data Availability

Not applicable/available for this study.
